# Risk Factors for Chronic Subdural Hematoma after a Minor Head Injury in the Elderly: A Population-Based Study

**DOI:** 10.1155/2014/218646

**Published:** 2014-09-11

**Authors:** Jen-Ho Tseng, Ming-Yuan Tseng, Ann-Jeng Liu, Wen-Hsiung Lin, Hsiao-Yun Hu, Sheng-Huang Hsiao

**Affiliations:** ^1^Department of Neurosurgery, Taipei City Hospital, Renai Branch, No. 4, Section 4, Renai Road, Taipei 106, Taiwan; ^2^Graduate Institute of Medical Sciences, College of Medicine, Taipei Medical University, No. 250, Wuxing Street, Taipei 110, Taiwan; ^3^Department of Education and Research, Taipei City Hospital, Renai Branch, No. 4, Section 4, Renai Road, Taipei 106, Taiwan; ^4^Institute of Public Health and Department of Public Health, National Yang-Ming University, No. 155, Section 2, Linong Street, Taipei 112, Taiwan; ^5^Institute of Traditional Medicine, National Yang-Ming University, No. 155, Section 2, Linong Street, Taipei 112, Taiwan

## Abstract

Chronic subdural hematoma (CSDH) is one of the major comorbidities in elderly resulting in disability and death. Early recognition of CSDH is important for early management. However, manifestations of CSDH are nonspecific and subtle. Therefore, identification of risk factors of CSDH can offer clinical follow-up strategies for patients after episodes of head injury. The purpose of the study aimed at identifying risk factors of CSDH of Taiwanese. Analysis of data from the National Health Insurance provides important information on predictive factors influencing the early diagnosis of CSDH in elderly patients following minor head injuries. The current study is the first nationwide population-based study in Taiwan, showing that old age (≥75 years), male gender, and coexisting hydrocephalus are significantly predictive factors, irrespective to their medical comorbidities.

## 1. Introduction

Chronic subdural hematoma (CSDH) is a relatively common affliction, especially among the elderly in whom the prevalence has been estimated to be 7.4~13.1/100,000 people [1, 2]. Although the diagnosis has been improved since the introduction of computerized tomography (CT), either many cases in the elderly aged above 65 years are missed or the diagnosis is delayed, due to nonfocal neurological deficits and/or concomitant chronic medical conditions. Indeed, it has been shown that CSDH in the elderly is more likely to be presented as mental changes instead of typical symptoms of increased intracranial pressure [3]. Miranda et al., based on a single center data, suggest that CSDH could be considered as a surrogate marker for underlying chronic diseases, which explains an accelerated mortality despite initial successful treatment [4]. Given a high concordance between the clinical and the National Health Insurance (NHI) data in Taiwan with over 95% of case coverage, this case-control study seeks to verify the above hypothesis by examining comorbidities in the elderly after a minor head injury at a national population level. Findings could be used for identifying the high risk population who can further benefit from early alerts and prompt treatment for CSDH.

## 2. Materials and Methods

This is a case-control study seeking to verify the hypothesis raised by Miranda et al. that CSDH may represent a surrogate marker for comorbidities in the elderly after a minor head injury. For better generalization of the study result, population-based data instead of individual hospital records are used.

### 2.1. Data

This anonymous dataset is extracted from the NHI in Taiwan as part of clinical audit, and therefore ethical approval is not required. Sources for the clinical information include records of hospital inpatients, outpatients, or general practitioners. In 1995, Taiwan launched the one single-payer NHI scheme replacing previous separate workplace health insurance funds, which is overseen by the NHI agency of the central government, Department of Health. It is mandatory for the health care providers to submit clinical data to the NHI agency for reimbursement, and therefore the NHI has been collecting the data to produce national information on all clinical patterns since then. Files are linked to a person-based database, updated, and ascertained. The completeness of coverage has been estimated at 95% [5, 6]. In this study, based on the population data from NHI instead of reviewing individual medical records, minor head injury was defined as head injury and/or cerebral concussion without concomitant intracranial hemorrhage (intracerebral, epidural, subdural, and subarachnoid hemorrhage). CSDH was defined as subdural hematoma (SDH) after head injury “AND” requiring burr hole drainage (surgery code of NHI in Taiwan: 83038; op-code of ICD-9-CM: 01.12, 01.31).

The following ICD-9 codes were used for extracting data: head injury (854.00–854.19), cerebral concussion (850.0–850.9), subdural hematoma (432.1), diabetes mellitus (DM, 250.*X*), hypertension (401–405), chronic kidney disease (585), chronic liver disease (571.0–571.9), rheumatic heart disease (91, 392–398), ischemic heart disease (410–414), atrial fibrillation/atrial flutter (Af/AF, 427.31, 427.32), heart failure (428.0–428.4), peripheral artery occlusion (440.21, 440.23), deep vein thrombosis (451.11, 451.19, 453.41, 453.42, 453.9, 454.0, 454.1, 454.2, and 454.9), and hydrocephalus (331.3, 331.5). The comorbidities selected were considered to be associated with coagulopathy, long-term treatment by anticoagulants or antiplatelet agents, or potential intracranial hypotension by diversion therapy of cerebrospinal fluid (CSF). These comorbidities were potential risk factors of CSDH formation after minor head injury.

**Figure 1:**
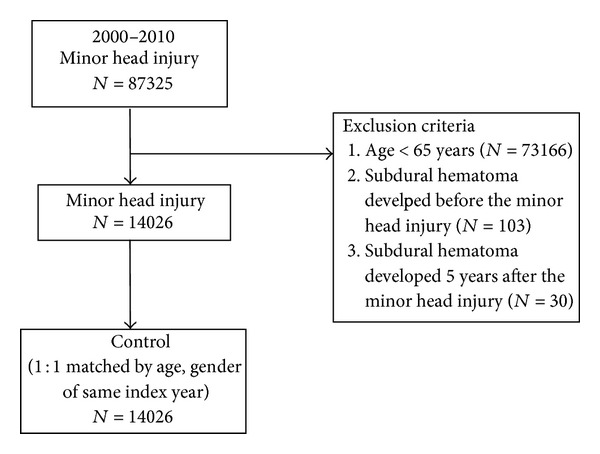
Flow chart of selection of cases and controls.

From 2000 to 2010 data of 87325 patients with a minor head injury admitted either as in- or outpatients are available in the NHI for analysis. For the purpose of analyzing potential predictive factors for developing CSDH after a minor head injury in the elderly, 73166 patients aged <65 years, 103 patients whose CSDH is diagnosed before minor head injury, and 30 patients with CSDH diagnosed 5 or more years following the minor head injury are excluded. A total of 14026 patients are finally eligible for analysis. For comparison, a control group of 14026 elderly was selected randomly from one of the individuals who matched the case's age and gender of the same index year.  Figure 1 illustrates the selection of cases and controls.

All the data were obtained under the permission and supervision of the Institution Review Board, Taipei City Hospital (TCHIRB-1020715-W).

### 2.2. Analysis

Analysis is performed by using SAS statistical package (SAS System for Windows, Version 8.2). Distributions among different variables are compared using *χ* ^2^ test. Multivariate analysis for the 11 comorbidities is performed using the Cox proportional hazards regression model. The hazards ratio (HR) with a 95% confidence interval (95% CI) expresses the probability of occurrence of CSDH in patients with a minor head injury relative to the control. The improvement in fit due to each variable is tested for statistical significance at the 5% level with the log likelihood ratio test. In these analyses, it is considered significant when the *P* value falls below 0.05.

## 3. Results

Table 1 shows the distribution of the 11 comorbidities in the 28052 adult patients. Patients aged >65 years with minor head injury are more likely to have had comorbidities, including DM (36.2% versus 32.1%, *P* < 0.001), hypertension (75.2% versus 70.2%, *P* < 0.001), chronic kidney disease (9.2% versus 7.4%, *P* < 0.001), chronic liver disease (24.9% versus 21.6%, *P* < 0.001), rheumatic heart disease (3.5% versus 2.7%, *P* < 0.001), ischemic heart disease (46.8% versus 40.3%, *P* < 0.001), Af/AF (5.3% versus 4.8%, *P* = 0.047), heart failure (19.1% versus 15.7%, *P* < 0.001), peripheral artery occlusion (0.5% versus 0.4%, *P* = 0.026), deep vein thrombosis (2.4% versus 2.0%, *P* = 0.028), and hydrocephalus (1.5% versus 0.5%, *P* < 0.001), and develop CSDH (1.8% versus 0.7%, *P* < 0.001).

Table 2 compares 347 elderly patients with CSDH versus 27705 elderly patients without SDH with respect to the comorbidities. Patients who have developed CSDH are more likely to be male (65.7% versus 49.7%, *P* < 0.001), aged above 75 years (55.6% versus 46.5%, *P* < 0.001), and have had hydrocephalus (3.5% versus 1.0%, *P* < 0.001) and a history of minor head injury (73.2% versus 49.7%, *P* < 0.001).

Table 3 shows the Cox regression model for predictors of CSDH development among patients with a minor head injury versus those without minor head injury. Because, among the patients with no SDH, none have had peripheral artery occlusion, this comorbidity is therefore removed from the modelling. Patients who have developed SDH following minor head injury are likely to be male (HR 1.96, 95% CI 1.57–2.45, *P* < 0.001), aged above 75 years (HR 1.68, 95% CI 1.35–2.08, *P* < 0.001), or have had hydrocephalus (HR 2.57, 95% CI 1.44–4.58, *P* < 0.001). They are less likely to have chronic kidney disease (HR 0.62, 95% 0.39–0.98, *P* = 0.042).

Table 4 shows the Cox regression model for predictors of CSDH development in patients with a minor head injury versus those without minor head injury, stratified by age (≤75 versus >75 years). Among the patients aged ≤75 years, those who have developed CSDH following minor head injury are likely to be male (HR 1.66, 95% 1.20–2.31, *P* = 0.002) but are less likely to have ischemic heart disease (HR 0.57, 95% 0.39–0.83, *P* = 0.004). However, among the patients aged >75 years, those who have developed SDH following minor head injury are likely to be male (HR 2.25, 95% CI 1.66–3.05, *P* < 0.001) or have had hydrocephalus (HR 2.76, 95% CI 1.41–5.41, *P* = 0.003) but are less likely to have chronic kidney disease (HR 0.51, 95% 0.27–0.98, *P* = 0.042).

## 4. Discussion

Results from this study are consistent with previous reports that the occurrence of CSDH is associated with old age, male gender, and history of head injury [7–9]. However, we also have identified that hydrocephalus is another significant risk factor; patients who have had hydrocephalus are two to three times more likely to develop CSDH following a minor head injury. This feature is particularly apparent among those whose age is above 75 years. Although it is not possible to differentiate the subtypes of hydrocephalus by using the ICD-9 codes (331.3, 331.5), the hydrocephalus in the elderly is most likely to be of normal pressure [10], and the diagnostic codes may also imply radiologic features of brain atrophy. The association between hydrocephalus and CSDH could be therefore due to a complication of shunting procedures for normal pressure hydrocephalus (NPH) [11] and a consequence of vulnerable bridging veins in elder-associated brain atrophy.

Restricted by the ICD-9 code 432.1 for subdural hematoma, it is not possible to differentiate acute versus chronic. However, given that acute subdural hematoma is associated with major head trauma and more likely to occur in younger patients, we believe that, by excluding patients aged <65 years from those who have had a minor head injury, the subdural hematoma in this dataset should represent CSDH. Further, for excluding spontaneous CSDH, the occurrence of CSDH before the minor head injury or five years and above after the minor head injury is excluded. Although the prevalence of CSDH in the control group is 0.7%, that is, 100 times of the reported prevalence as 7.4/100,000 [1], our study showed that the data does not represent the general population and only included CSDH after minor head injury “AND” requiring surgery. The CSDH in patients who are admitted as in- or outpatients without precipitated head injuries is most likely to be associated with spontaneous occurrence or an obscured history of minor head injuries [3] that cannot be included in our study.

We have chosen DM, hypertension, chronic kidney disease, chronic liver disease, ischemic heart disease, and heart failure as common medical conditions for hospital admissions in Taiwan. We also have chosen rheumatic heart disease, Af/AF, peripheral artery disease, and deep vein thrombosis, on the basis of required anticoagulant therapy, representing subsequent pharmacological coagulopathy, another known risk factor for developing CSDH [7].

The univariate analyses show that patients aged >65 years who have had minor head injury are more likely to have had comorbidities and developed CSDH. However, only male gender, old age (>75 years), and hydrocephalus are predictive factors for developing CSDH in this population demonstrated by the multivariate Cox regression modelling. The chronic kidney disease seems to be a protective factor for the occurrence of CSDH, probably because the patient's mobility is restricted by the renal replacement therapy or patient's own understanding of the disease, which may have a limiting effect on the severity of minor head injuries. Likewise, the potential protective effect from the ischemic heart disease could be associated with a reduction in patient mobilization.

Although the data from the NHI does not contain information on treatment modalities and functional outcome, we believe that the information extracted can help identify most vulnerable patients, design potential treatment modalities, and amend health policies. On the basis of our nationwide data, we do not agree with Miranda et al. that CSDH should be considered as a surrogate marker for underlying chronic medical conditions [4]. Instead, our data suggests that patients who are male elderly with shunted NPH are more likely to develop CSDH following a minor head injury, irrespective of their comorbidities. These patients should have frequent neurological and/or imaging followup. Furthermore, both the patients and their caregivers should be alerted to the potential development of CSDH.

## 5. Conclusion

Analysis of data from the NHI provides important information on predictive factors influencing the early diagnosis of CSDH in elderly patients following minor head injuries. The current study is the first nationwide population-based study in Taiwan, showing that old age, male gender, and coexisting hydrocephalus are significantly predictive factors. Results from this population-based study are very helpful for comparison with those of other hospital-based studies and for public health purposes. Therefore, for more cost-effective allocation of health resources, the clinician may consider following up this high risk population more frequently after their minor head injuries and both the patients and their caregivers should be well informed of the potential development of CSDH.

## Figures and Tables

**Table 1:** Baseline characteristics of the study subjects.

Variables	Patients with minor head injury		Control		*P* value
	*n* = 14026	%	*n* = 14026	%	
Gender					1.000
Female	7023	50.1	7023	50.1	
Male	7003	49.9	7003	49.9	
Age					0.416
≤75	7,456	53.2	7,524	53.6	
>75	6,570	46.8	6,502	46.4	
Comorbidity					
DM	5,074	36.2	4,496	32.1	<0.001
Hypertension	10,550	75.2	9,852	70.2	<0.001
Chronic kidney disease	1,296	9.2	1,038	7.4	<0.001
Chronic liver disease	3,495	24.9	3,024	21.6	<0.001
Rheumatic heart disease	489	3.5	380	2.7	<0.001
Ischemic heart disease	6,571	46.8	5,647	40.3	<0.001
Af/AF	749	5.3	676	4.8	0.047
Heart failure	2,677	19.1	2,206	15.7	<0.001
Peripheral artery occlusion	76	0.5	51	0.4	0.026
Deep vein thrombosis	337	2.4	283	2.0	0.028
Hydrocephalus	214	1.5	75	0.5	<0.001
Subdural hematoma	254	1.8	93	0.7	<0.001
Followup year (mean ± SD)	4.39 ± 3.18		4.68 ± 3.15		

**Table 2:** Baseline characteristics of the study subjects.

Variables	Subdural hematoma		No subdural hematoma		*P* value
	*n* = 347	%	*n* = 27705	%	
Gender					<0.001
Female	119	34.3	13927	50.3	
Male	228	65.7	13778	49.7	
Age					<0.001
≤75	154	44.4	14,826	53.5	
>75	193	55.6	12,879	46.5	
Comorbidity					
DM	120	34.6	9,450	34.1	0.854
Hypertension	245	70.6	20,157	72.8	0.371
Chronic kidney disease	19	5.5	2,315	8.4	0.054
Chronic liver disease	64	18.4	6,455	23.3	0.033
Rheumatic heart disease	10	2.9	859	3.1	0.815
Ischemic heart disease	141	40.6	12,077	43.6	0.270
Af/AF	12	3.5	1,413	5.1	0.166
Heart failure	4,831	1,392.2	52	0.2	0.231
Peripheral artery occlusion	127	36.6	0	0.0	0.413
Deep vein thrombosis	6	1.7	614	2.2	0.540
Hydrocephalus	12	3.5	277	1.0	<0.001
Minor head injury	254	73.2	13,772	49.7	<0.001

**Table 3:** Cox regression model for predictor of subdural hematoma development.

Variables	HR	95% CI	*P* value
Gender			
Female	1.00		
Male	1.96	1.57–2.45	<0.001
Age			
≤75	1.00		
>75	1.68	1.35–2.08	<0.001
Comorbidity			
DM	1.18	0.93–1.48	0.173
Hypertension	0.91	0.71–1.16	0.443
Chronic kidney disease	0.62	0.39–0.98	0.042
Chronic liver disease	0.76	0.58–1.00	0.052
Rheumatic heart disease	1.03	0.55–1.96	0.917
Ischemic heart disease	0.89	0.70–1.12	0.312
Af/AF	0.68	0.38–1.22	0.196
Heart failure	0.86	0.63–1.18	0.345
Deep vein thrombosis	0.79	0.35–1.78	0.572
Hydrocephalus	2.57	1.44–4.58	0.001

**Table 4:** Cox regression for subdural hematoma among patients with minor head injury and controls, by age group.

Variables	Age ≤ 75			Age > 75		
	HR	95% CI	*P* value	HR	95% CI	*P* value
Gender						
Female	1.00			1.00		
Male	1.66	1.20–2.31	0.002	2.25	1.66–3.05	<0.001
Comorbidity						
DM	1.24	0.88–1.75	0.221	1.13	0.83–1.55	0.438
Hypertension	0.94	0.65–1.35	0.741	0.88	0.63–1.25	0.476
Chronic kidney disease	0.78	0.39–1.55	0.484	0.51	0.27–0.98	0.042
Chronic liver disease	0.79	0.54–1.16	0.230	0.73	0.49–1.09	0.121
Rheumatic heart disease	1.24	0.45–3.42	0.682	0.92	0.40–2.09	0.839
Ischemic heart disease	0.57	0.39–0.83	0.004	1.21	0.89–1.65	0.224
Af/AF	0.72	0.26–1.98	0.519	0.66	0.32–1.37	0.267
Heart failure	0.94	0.55–1.61	0.815	0.81	0.55–1.19	0.289
Deep vein thrombosis	0.63	0.16–2.56	0.523	0.91	0.34–2.46	0.852
Hydrocephalus	2.04	0.65–6.43	0.223	2.76	1.41–5.41	0.003

## Abbreviations

95% CI: 95% confidence interval
CSDH: Chronic subdural hematoma
DM: Diabetes mellitus
HR: Hazards ratio
ICD-9: International Classification of Diseases 9th Edition
NHI: National Health Insurance.
